# Patterns of limitations in activities of daily living, sleep, and pain in the early postoperative period following total shoulder arthroplasty: a prospective study

**DOI:** 10.1016/j.jseint.2022.09.017

**Published:** 2022-11-01

**Authors:** Oluwadamilola Kolade, Niloy Ghosh, Daniel Buchalter, Yoav Rosenthal, Joseph D. Zuckerman, Mandeep S. Virk

**Affiliations:** Division of Shoulder & Elbow, Department of Orthopaedic Surgery, NYU Langone Orthopedic Hospital, New York, NY, USA

**Keywords:** Activities of daily living, Total shoulder arthroplasty, Reverse shoulder arthroplasty, Shoulder pain, Sleep disturbances, Visual analog scale

## Abstract

**Background:**

The aim of this study is to investigate the pattern of changes in activities of daily living (ADLs), sleep disturbance, and pain in the early postoperative period following a total shoulder arthroplasty (TSA).

**Methods:**

Prospective data on patterns of limitation in ADLs, sleep disturbance, and pain were collected from patients undergoing elective TSA preoperatively and at specific time points postoperatively (2, 6, and 12 weeks). At each time point, patients were asked regarding the major limitation affecting their shoulder. Limitations in ADLs and sleep disturbances were scored on a 3-point scale (0 = unable to do, 3 = no difficulty) modeled after the ADL which require active external rotation score and visual analog scale scores were used for pain. Patient responses were analyzed with respect to patient factors (demographics, arm dominance, function of opposite arm, and ambulation status), and living situation (alone, or with caregiver).

**Results:**

Shoulder pain (43%) and inability to perform ADLs (38%) were the 2 most commonly reported limitations prior to undergoing TSA. Patients noticed progressive improvements in pain with 37% reductions in visual analog scale scores at 2 weeks and 67% reduction at 3 months. At 2 weeks after TSA, sleep disturbances were the most disabling issue in 33% of the cohort, with considerable improvements (104%) in sleep scores at 3 months compared to pre-op. The ADLs involving forward elevation and working at the waist level improved considerably between 6 weeks and 3 months, but activities involving rotation including reaching behind the back, across the chest, and use of strength showed mild improvements by 3 months.

**Conclusion:**

This prospective study demonstrates the chronology of improvements in pattern of limitations experienced by patients with respect to pain, sleep, and ADLs in the early postoperative period after TSA. Majority of patients can expect to have 2/3 resolution of pain, improved sleep, and improvement in ADLs involving forward elevation and waist level function by 3 months.

Total shoulder arthroplasty (TSA) is a successful surgical treatment for end stage primary and secondary shoulder arthritis. The primary objectives of shoulder arthroplasty are to reduce pain, restore function, and improve the quality of life. Numerous studies have demonstrated long-term improvements in these parameters compared to baseline (preoperative) after TSA.^3^^,^^6^

Although short-, mid-, and long-term outcomes of TSA have been extensively published, there is limited literature on the chronological pattern and extent of limitations incurred with respect to postoperative pain, sleep disturbances, and activities of daily living (ADLs) limitations in first few months after TSA. There are substantial dynamic improvements and changes that occur during the first several weeks after TSA with respect to pain relief, sleep cycle changes, and ability to perform ADLs. During this time patient’s interaction with the surgeon are more frequent and questions regarding recovery are more common. Although improvements in outcomes scores and pain scores during the early postoperative period are helpful for patients to understand the big picture of their recovery, it has been our observation that information about chronology of improvements in pain and function are lacking. Prior studies have focused on characterizing improvements in shoulder specific outcomes after TSA but these outcome scores do not take into consideration the important limitations specific to ADLs, sleep disturbances and extent of pain relief in the first several weeks after shoulder arthroplasty.^1^^,^^5^^,^^10^^,^^11^ Therefore, the purpose of this study is to prospectively examine the pattern of self-reported improvements (chronological, and objective) with respect to ADLs, sleep changes, and pain in the early postoperative period (first 3 months) after TSA.

## Methods

In this prospective study from September 2018 to May 2019, consecutive patients who underwent TSA (anatomic or reverse) by 1 of 3 fellowship trained orthopedic surgeons were included in the study. Patients were included in the study if they were over 18 years of age, had undergone either anatomic or reverse TSA (rTSA), and have follow-up visits at the 2 weeks, 6 weeks, and 3 months. Patients were excluded if the arthroplasty indication was acute fracture, or if they were unable or unwilling to complete the study questionnaire.

Each patient gave informed consent and completed a questionnaire preoperatively and at the 2-week, 6-week, and 3-month postoperative time points assessing the level of daily and athletic activity, sleep disturbances, and level of pain following shoulder arthroplasty. The questionnaire included demographics (gender, marital status, race, ethnicity, employment status, occupation, yearly income, and living situation), pain scores, operative information, and questions regarding ADLs. These included clothing related activities (5 questions), personal hygiene activities (5 questions), food preparation (4 questions), household activities (4 questions), general mobility (4 questions), and general activities (3 questions). Patient responses to the ADLs questions were scored on a 3-point scale ranging from a lower functioning (0 = unable to do) to a higher functioning (3 = no difficulty) level modeled after the ADL which require active external rotation score.^2^^,^^4^ A total of 25 different activities were assessed, leading to a maximum possible score of 75 points. Sleep disturbances were grouped into one or more of the following: difficulty in falling sleep, interrupted sleep, and reduced sleep time and scored on a 3-point scale ranging from a 0 (unable to do) to 3 (no difficulty) level. The patients also self-identified the most significant limitation at each time point starting from preoperatively and postoperatively at 2 weeks, 6 weeks, and 3 months. All data from the questionnaires were collected and stored securely using REDCap (Vanderbilt University; Nashville, TN, USA).

Response data were tabulated for the entire study group including both numerical data and respondent written answers. Data were divided into subgroups based on time of follow-up at 2 weeks, 6 weeks, or 3 months. The data was further subdivided categorically based both on the aforementioned activity groups in the ADLs as well as by primary motion required (forward flexion, abduction, extension, external rotation, and internal rotation). Additional subdivisions were made dependent on whether the patient underwent an anatomic TSA or an rTSA.

### Surgical details

During the defined study period patients with primary or secondary arthritis who were indicated for TSA (anatomic or reverse) were included in the study. All patients underwent TSA via a deltopectoral approach. In the anatomic TSA (aTSA), subscapularis tenotomy was performed in all cases and repaired at the end of the procedure. Three different anatomic implant types (DJO Inc., Vista, CA, USA; Exactech Inc. Gainesville, FL, USA; and Tornier Inc., Bloomington, MN, USA) were used by the surgeons. For rTSA, subscapularis tenotomy was performed when subscapularis was intact or partially torn. The tenotomy was repaired at the end of procedure unless it was not reparable. Both inlay (DJO Inc.) and onlay (Exactech Inc. and Tornier Inc.) designs implants were used by the surgeons.

### Postoperative immobilization and rehabilitation

The arm was placed in a sling for 4-6 weeks in patients that underwent aTSA. For rTSA the sling use varied as per surgeon’s preference (2 weeks vs. 6 weeks). Physical therapy was started on all TSAs on day 1 after surgery. TSA precautions were followed in a similar fashion for all shoulder arthroplasties for at least 6 weeks

In this study the primary outcome of interest is the pattern of restoration of the ability to complete ADLs following TSA. This was represented using 2 novel clinical outcome indexes, the total ADLs score and the percentage adjusted ADLs score. Subgroup analysis was performed to assess patient’s ability to perform a subset of activity type, giving insight on when patient can return to normal function. The statistical analysis was performed using R studio (R Studio; Boston, MA, USA).

## Results

### Patient characteristics

This study included a total of 154 patients, all of whom met the aforementioned inclusion and exclusion criteria. Of these patients, 154 completed the preoperative survey, 105 completed the 2-week survey, 105 completed the 6-week survey, and 101 completed the 3-month survey. The cohort was split almost evenly by gender (51% female, 49% male), and 60% were retired (Table I). The patients’ mean age was 67 years (Standard Deviation, 10.0 years; Range, 38-88 years).

**Table I:** Patient demographic characteristics (total cohort, n = 154).

Parameter	Value
Gender	
Male	49%
Female	51%
Arm operated on	
Dominant	54%
Non-dominant	46%
Age	
<30	0%
31-40	1%
41-50	5%
51-60	10%
61-70	45%
71-80	30%
>80	9%
Race	
White	91%
Black/African American	5%
Asian/Pacific Islander	0%
Native American	1%
More than one Race	1%
Unknown/not reported	2%
Ethnicity	
Hispanic or Latino	9%
Not Hispanic or Latino	83%
Unknown/not reported	8%
Marital Status	
Single or never married	19%
Married	58%
Divorced/separated	11%
Widowed	10%
Separated	2%
Employment status	
Full-time employment	25%
Part-time employment	4%
Unemployed	6%
Self-employed	7%
Homemaker	1%
Student	0%
Retired	57%
Current living situation	
No caregiver	77%
One or more caregivers	23%
Help required for daily activities	
Need for most/all activities	3%
Need for some activities	17%
Need for few activities	5%
Need for no activities	75%

The preoperative questionnaire was completed by 154 patients prior undergoing either aTSA or rTSA. Of the 105 patients that completed the 2 week postoperative questionnaire underwent 81 aTSA and 24 rTSA. There were no significant differences in preoperative ADL scores with respect to gender, (males [51.8] and females [43.8]) race, marital status, arm dominance, and ethnicity. Employment was one factor that showed to affect ADL scores. Patients who were retired and unemployed had a significantly lower ADL scores compared to patients who were employed either full time or part time, or self-employed. Patients in the lowest income bracket of less than 25,000 of annual income were shown to have statistical impactful lower ADL scores compared to patients with higher annual income (*P* = .03). Patients who were operated on their right arm (*P* = .05) and had higher preoperative visual analog scale (VAS) pain scores had significantly lower preoperative ADL scores (*P* = .03).

### Pattern of limitations of shoulder pain

**Figure 1:**
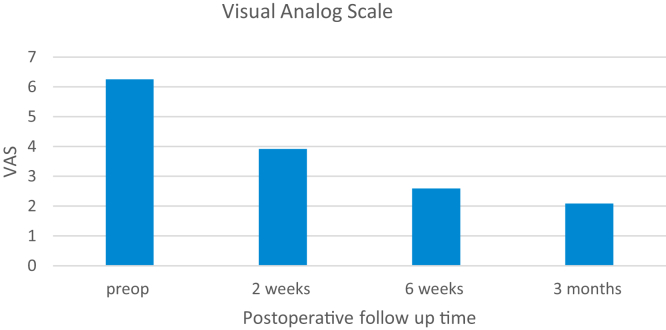
Bar graph demonstrating visual analog scale scores of patients at preoperatively and at each follow-up after total shoulder arthroplasty. *VAS*, visual analog scale.

Preoperatively pain was the most common complaint with 43.5% of patients reporting pain as the sole reason to undergo TSA. At the 2-week postoperative period there was 37% reduction in pain from preoperative baseline pain with only 16% patients reporting pain as most disabling symptoms. The VAS scores demonstrated 59% reduction at 6-week and 67% reduction at 3-month time point (Fig. 1)

### Pattern of limitations of sleep

**Figure 2:**
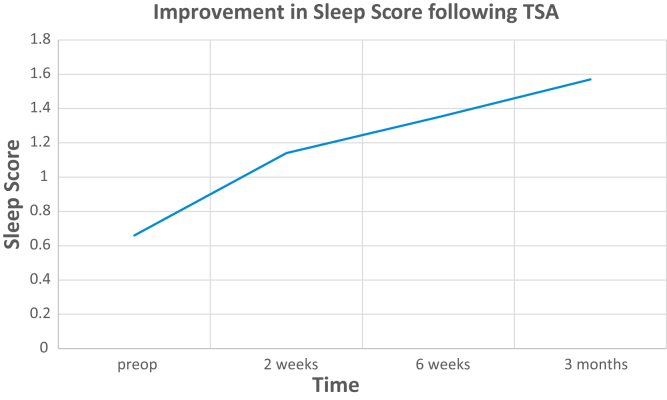
Line graph showing progression of sleep score of patients preoperatively and at each follow-up after total shoulder arthroplasty. *TSA*, total shoulder arthroplasty.

Preoperatively, 13% of the cohort complained of sleep disturbances as primary complaint, and this number actually peaked postoperatively to 33% at 2 weeks and subsequently decreased to 26% at 6 weeks and 21% of the cohort at 3 months. The sleep score demonstrated gradual improvement postoperatively compared to preoperative score with 72% improvement at 6-week time point and 104% improvement at 3-month time point (Fig. 2)

### Pattern of limitations of ADL

**Figure 3:**
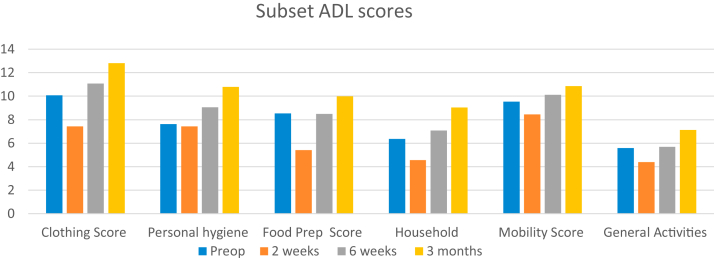
Bar graph showing progression of activities of daily living Subset scores of patients and at each follow-up after total shoulder arthroplasty. *ADLs*, activities of daily living.

Prior to surgery, approximately 39% of the patients reported inability to perform ADLs as the major complaint secondary to shoulder arthritis, and this proportion of cohort did not show any appreciable change at 2-week (5% reduction) and 6-week (3% reduction) after shoulder arthroplasty. However, there was a considerable improvement (34% reduction) in these limitations at 3 months after TSA. The mean ADL score improved to 61 at 3 months from a preoperative score of 47 (Fig. 3).

**Figure 4:**
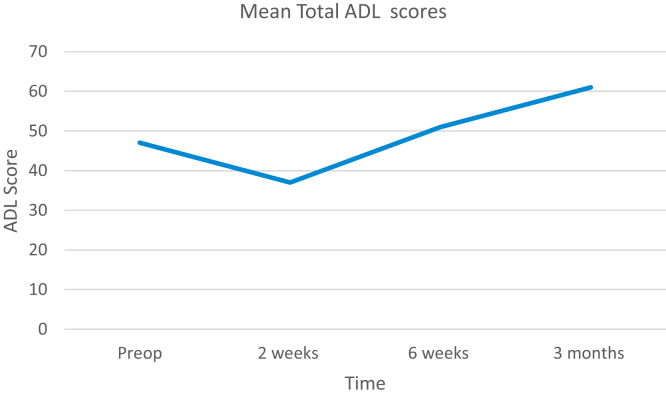
Line graph showing progression of the mean total activities of daily living score of patients preoperatively and at each follow-up after total shoulder arthroplasty. *ADLs*, activities of daily living.

The mean functional score improved from 1.59 preoperatively to 2.23 (70% increase) at 3 months, with specifically a 19% and 23% improvement in activities performed at work and while taking part in hobbies, respectively (Fig. 4).

The ADL scores related to clothing tasks that showed a similar trend with gradual improvement by 10% and 19% at 6 weeks and 3 months, respectively. The subgroup analysis demonstrated that most difficult task reported by the cohort was putting on a shirt with an average score of 1.70, which improved to 1.84 and 2.27 at 6 weeks and 3 months, respectively (Fig. 3).

The ADL scores related to personal hygiene showed a decrease at 2-week time point but there was considerable improvement in this score at the 6 weeks (18.7% improvement) and 3 months (41.2% improvement) time points. However, patients consistently demonstrated difficulty reaching the opposite shoulder/arm pit, and reaching behind the back during activities of personal hygiene at 3 months (Fig. 3).

The ADL scores related to the food preparation demonstrated a 36% reduction at 2 weeks after surgery compared to preoperative scores. At the 6-week time point, the majority of patients reported restoration to preoperative function and at 3 months there was 16.8% further improvement compared to preoperative scores. Opening a tight jar was the most difficult task reported by the cohort preoperatively and at 3 months (Fig. 3). The ADL scores focused on the ability of patients to complete household tasks demonstrated a 41% improvement at 3 months. Reaching up to a high shelf was reported as the task with most limitation in the forward plane of the shoulder preoperatively with moderate overall improvement in this task at 3 months (Fig. 3). With respect to the ADLs related to general mobility, getting out of bed or chair and going up and down the stairs showed minimal limitations preoperatively and postoperatively (Fig. 3).

## Discussion

In this prospective cohort study, we describe the chronology of improvements that occur with regards to pain, sleep disturbances, and performing ADLs in the first three months following a TSA. We found that pain with or without limitations in ADLs was the most common preoperative reason to undergo a shoulder arthroplasty. Postoperatively, patients can expect a 2/3 reduction in their pain in first 3 months of recovery with some residual sleep disturbances at 3 months. The ADLs involving forward elevation (reaching shoulder level shelf in the forward plane), general mobility, and working at the waist level (food preparation) improves considerably at 3 months but activities involving rotation including reaching behind the back and across the chest, and activities involving use of strength show mild improvements by 3 months.

TSA has shown good-excellent short- and long-term outcomes for treatment of shoulder arthritis with considerable improvements in pain and functional outcome scores. There are considerable improvements that occur during the first several weeks after TSA with respect to pain relief, sleep cycle changes, and ability to perform ADLs, but chronology and frequency of these limitation and improvements are less well described in literature. In this prospective study, we investigated the challenges experienced by patients during first 12 weeks after TSA. We believe that this data will serve as a resource for counseling patients, as to what to expect in first 3 months after TSA.

It is important to note that chronology and extent of pattern of limitations after shoulder arthroplasty are not identical compared to after rotator cuff repair or instability repair even though the sling time may be similar. Postoperative pain levels after arthroscopic instability repair are less than those undergoing a shoulder arthroplasty. Although patients with arthroplasty have early onset of range of motion (ROM) exercises, the pattern of limitations and its recovery with respect to ROM and ADLs are likely to be different between post arthroplasty and rotator cuff repair or instability repair. A more objective insight into the aforementioned differences will require additional study including postsurgical patients with different surgical procedures

Shoulder pain was reported as the most common reason and limited function as the second most common reason to undergo a shoulder arthroplasty by our study cohort. Previous studies have demonstrated that TSA is a predictable operation for pain relief. Tashjian et al reported a 5.9-fold decrease in pre- and postoperative VAS scores following TSA, which was statistically significant.^14^ In this study they showed that the mean clinically important difference showcasing significant improvements in pain (mean clinically important difference VAS of 1.4) occurring as early as 2 weeks. Similarly, Simovitch et al corroborated that following shoulder arthroplasty patients had significant reduction in pain at the latest follow-up of 2 years following shoulder arthroplasty.^13^ Unlike these other studies, they did not follow progression of pain relief, which allows physician to guide patient’s expectations following shoulder arthroplasty. These other studies also do not discuss the pattern of pain relief in the acute postoperative period, which provides better guidance for physicians to manage immediate postoperative shoulder arthroplasty patients. Based on the data from our study, it is reasonable to tell the patients that pain levels will considerably decrease 63% in our study in the first two weeks after surgery.

Shoulder pathology has been correlated to have significant impact on sleep quality, sleep duration, and habitual sleep efficiency.^9^ Although TSA has shown to lead to resolution of sleep disturbances and improved quality of life, sleep disturbances are common within the first several weeks after TSA. Sleep disturbances have been attributed to a positional requirement, inability to sleep in the preferred position and temperature cha nges.^7^ Weinberg et al showed a progressive improvement of sleep quality after TSA, which returned to normal limits at 12 months after surgery.^15^ In this study, we demonstrate that sleep disturbances peak before 6 weeks and are much less thereafter, which could reflect the association of sleep disturbances with sling use and restrictions related to positioning of the arm.

Limitations in ROM following shoulder arthroplasty is a reasonable concern of patients after TSA. Although patients with shoulder arthritis have limited function prior to surgery, sling use in first 4-6 weeks after surgery makes the patient one handed for most part. Our study demonstrates that function involving forward elevation improves much faster than ADLs that required internal rotation behind the back or reaching across the body. ADLs involving rotation behind the back remain quite limited for a longer period of time due to the pattern of recovery of ROM after shoulder surgery and also in part due to restrictions imposed by the surgeons. Razmjou et al showcased statistically significant improvement of ROM at the 6-month postoperative mark, which continued to the 2-year postoperative follow-up compared to preoperative baseline.^12^ Kasten et al findings also corroborated improvement in completing ADLs at the 6-month postoperative follow-up following shoulder arthroplasty and showed a limitation in the reconstitution of some planes of shoulder motion.^8^ Kasten et al and Razmjou et al did not comment on improvements in the early postoperative phase, which is provided in our study.^8^^,^^12^ This is important information that can be shared with the patients in the postoperative period.

The construct of this study has several strengths and limitations. In this study we focused on the chronological sequence of presentation of the 3 most important limitations (as determined by the patients) and were able to determine the frequency and progression of the extent of improvement of these limitations at three time points (2 weeks/6 weeks/3 months). Study strengths include a large cohort, prospective standardized data collection, and a prior categorization of primary outcomes, and presentation of important clinical improvements with regards to ADLs, sleep, and pain management. In addition, there was limited recall bias because the data collection was on regular scheduled bias. However, there are also several important limitations. First, the sleep disturbances were subjectively determined and not objectively assessed. Second, the study duration was short (3 months) but because prior studies have reported on one-year outcomes, we wanted the focus of this study on first 3 months. Third, we did not have enough numbers to evaluate the differences in pattern of recovery in patients undergoing aTSA or rTSA. Finally, we did not have adequate control groups with respect to shoulder pathology (inflammatory arthritis, cuff tear arthropathy, and primary osteoarthritis) to determine differences in pattern of recovery in early phases after TSA.

## Conclusion

This prospective study demonstrates the chronology of improvements in pattern of limitations experienced by patients with respect to pain, sleep, and ADLs in the early postoperative period after TSA. The majority of patients can except to have 2/3 resolution of pain, improved sleep, and improvement in ADLs involving forward elevation and waist level function by 3 months.

## Disclaimers

Funding: The authors received no financial support for the research, authorship, and/or publication of this article.

Conflicts of interest: The authors, their immediate families, and any research foundation with which they are affiliated have not received any financial payments or other benefits from any commercial entity related to the subject of this article.
